# We should have seen this coming

**DOI:** 10.3389/fnhum.2014.00332

**Published:** 2014-05-27

**Authors:** D. Samuel Schwarzkopf

**Affiliations:** Experimental Psychology, University College LondonLondon, UK

**Keywords:** scientific method, falsifiability, Parsimony, precognition, presentiment

The possibility of precognition has fascinated humanity since ancient times making it a recurring theme in fiction and mythology. It has also been a topic for scientific investigation. While the majority of such parapsychological studies have been ignored by the larger scientific community, several recent studies of purported precognitive phenomena were published by major international psychology journals. A widely publicized study by Daryl Bem claimed to have found evidence of precognition (Bem, 2011). In its wake have been discussions about the appropriate statistical approach for testing these effects (Bem et al., 2011; Rouder and Morey, 2011; Wagenmakers et al., 2011), and it caused a wave of replication attempts most of which, at least those conducted by researchers skeptical of precognition, have failed (Galak et al., 2012; Ritchie et al., 2012; Wagenmakers et al., 2012). More recently, two articles in *Frontiers in Psychology* and * Frontiers in Human Neuroscience* reported a meta-analysis of experiments on “predictive anticipatory activity” or “presentiment” (Mossbridge et al., 2012, 2014). In that paradigm participants are exposed to a series of random stimuli, some arousing (violent/erotic images, loud sounds), others calm controls (neutral images, silence). Apparently, physiological responses evoked by the two trial types *prior to stimulus onset* predict the upcoming stimulus.

Such findings of “psi” effects fuel the imagination and most people probably agree that there are things that current scientific knowledge cannot explain. However, the seismic nature of these claims cannot be overstated: future events influencing the past breaks the second law of thermodynamics. If one accepts these claims to be true, one should also be prepared to accept the existence of perpetual motion and time travel. It also completely undermines over a century of experimental research based on the assumption that causes precede effects. Differences in pre-stimulus activity would invalidate baseline correction procedures fundamental to many different types of data analysis. While the meta-analysis briefly discusses this implication (Mossbridge et al., 2012), the authors are seemingly unaware of the far-reaching consequences of their claims: they effectively invalidate most of the neuroscience and psychology literature, from electrophysiology and neuroimaging to temporal effects found in psychophysical research. Thus, it seems justified to ask for extraordinary evidence to support claims of this magnitude (Truzzi, 1978; Sagan, 1995).

But what constitutes extraordinary evidence? The results of this and other similar meta-analyses on psi effects are highly significant under commonly used inferential statistics and in many cases also strongly supported by Bayesian inference. Applying the standards accepted by the larger scientific community, should this not suffice to convince us that precognition/presentiment are real?

To me this interpretation betrays a deep-seated misapprehension of the scientific method. Statistical inference, regardless of whatever form it takes, only assigns probabilities. It cannot ever prove or disprove a theory. In fact, unlike mathematical theorems, scientific theories are never proven. They can only be *supported* by evidence and must always be subjected to *scientific skepticism*. The presentiment meta-analysis (Mossbridge et al., 2012, 2014) illustrates how this process can be misapplied. A significant effect does not confirm psi but it raises many new questions.

First, any meta-analysis can only be as good as the primary studies it analyzes. Several of the studies included are of questionable quality, e.g., the fMRI experiment (Bierman and Scholte, 2002) commits major errors with multiple comparison correction and circular inference (Kriegeskorte et al., 2009; Vul et al., 2009) and has such poor presentation that it is unlikely it would have been accepted for publication in any major neuroimaging journal. Moreover, many studies were in fact published in conference proceedings and did not pass formal peer review. Admittedly, the authors go to some lengths to assess the quality of each study but it is unclear how appropriate those quality scores were. In fact the rationale for the formula used to combine the different measures is debatable. A more detailed breakdown of how these different parameters influence the results would have been far more interesting. Does the type of random number generator used, or whether a study was peer reviewed, make any difference to the results? Related to this, additional factors would have been of importance, such as whether the experimenters expected to find a presentiment effect (see also: Galak et al., 2012).

Second, the meta-analysis should be much broader including myriad studies not conducted by psi researchers that used similar designs. While the authors tested for potential publication bias (the possibility that many null-results that would have made the results non-significant are missing from the database), there must be a large number of data sets from similar protocols in the wider literature, in particular in emotion research, whose inclusion would greatly enhance the power of this analysis. An often used argument, that these studies are invalid because they used counterbalanced designs and are thus confounded by expectation bias, is a straw man and also rather ironic given the topic of investigation: unless participants *knew in advance* that stimuli were counterbalanced this could not possibly change their expectations.

Third, a particular critical factor that should have been analyzed directly is the imbalance between control (calm) and target (arousing) trials typically used in these studies. While the authors themselves acknowledge that this is usually approximately 2:1 (Mossbridge et al., 2012), this is neglected by the meta-analysis and seemingly most primary studies even though such an imbalance means that an attentive participant will quickly learn statistical properties of the sequence and thus affects how the brain responds to the different stimulus classes. Rather than predicting future events, what such pre-stimulus physiological activity may actually reflect is that the brain can *make predictions of probable events*. One important factor to be included in the meta-analysis therefore should have been whether the ratio of target and control trials affects the magnitude of these pre-stimulus effects.

Fourth, could these effects be at least partially explained by analytical artifacts? In many of these studies (Bierman and Scholte, 2002; Radin, 2004) the data are not only baseline corrected to the mean activity level prior to stimulus onset, but they are further “clamped” to a particular time point prior to the stimulus. This should not necessarily influence the results if this point is a true baseline. However, if this pre-stimulus period is still affected by the response to the previous stimulus (e.g., the signal could decay back to baseline more slowly after an arousing than a calm trial) such a correction would inadvertently introduce artifacts in the pre-stimulus period. As such it may also be a much greater problem for slow than fast physiological measures. Either way, it would have been another factor worthy of attention in the meta-analysis.

Fifth, the effect of expectation and trial order must be tested explicitly. Many of these studies test for the presence of expectation bias by correlating the presentiment effect with the time between target events (Mossbridge et al., 2012). The rationale is that expectation bias increases with the number of control trials—a gambler's fallacy. Therefore, so the reasoning goes, the pre-stimulus activity should also increase. However, this is based on an unproven assumption that these physiological effects scale linearly with expectation. Further, because the probability of sequences of control trials falls off exponentially with their length, the presentiment effect cannot be estimated with the same reliability for long sequences as for short ones. This means that even if a linear relationship exists, the power to detect it is low as the presentiment response is subject to variability. Critically, this analysis cannot possibly detect whether participants learn statistical regularities in the trial sequence. The best approach to understand the role of trial order and expectation would be to design experiments that directly test these factors. Order effects are quantified by exposing participants to different stimulus pairs. Expectation bias can be manipulated by cuing participants to the probability that the upcoming trial is a target. If the predictive activity were similar to the presentiment effect when participants strongly expect a target trial, this would indicate that it may in fact be an expectation effect. Crucially, does presentiment persist when participants *do not expect* a target even when the next trial is one?

Lastly, are the purported effects even biologically plausible? These studies employ vastly different measurements from skin conductance and electrophysiology to hemodynamic responses. Under conventional knowledge these are assumed to be caused by preceding neural events, e.g., a typical hemodynamic response peaks ~6 s after a neural event (Boynton et al., 1996). Conversely, electrophysiological measures have a latency of fractions of a second, while skin conductance measures, heart rates, pupil dilation etc. probably fall somewhere in between. Thus, the same precognitive neural event probably cannot cause all of these responses. Alternatively, if these responses themselves reverse the arrow of time and are caused by future stimuli, this will require a complete overhaul of current theory. Why should blood oxygenation increase before neural activity in such a way that predicts the up-coming stimulus?

In my mind, only if all these points were addressed appropriately, should one even entertain the thought that such presentiment effects have been empirically demonstrated. The first four could have certainly formed part of the meta-analysis; it is frankly a clear failure of the peer review process that they were not. The final two points should at the very least be discussed as critical further steps before assuming that the effects reverse causality.

Much of parapsychology research is concerned with proving that psi is real (Alcock, 2003), that is, it rests on the notion that “there is an anomalous effect in need of an explanation” (Utts, 1991). But there is always *unexplained variance* in any data regardless of how significant the results are. It is the purpose of scientific investigation to explain as much as possible, not to conclude that there remains something we do not currently understand. Science works by formulating *falsifiable hypotheses* and testing them. This in turn will produce new findings that can be used to generate better theories. Further, one should always start from the *most parsimonious explanation* for a result. Only if a more complex model has greater *explanatory power* should we stray from the one requiring the least assumptions (Figure 1). Since the psi hypothesis merely postulates that some results remain unexplained, it does not lead to any falsifiable hypothesis that could help explain these effects. *Post-hoc* speculations of quantum biology or psychokinesis are insufficient unless they can make testable predictions and they are hardly the most parsimonious explanations.

**Figure 1:**
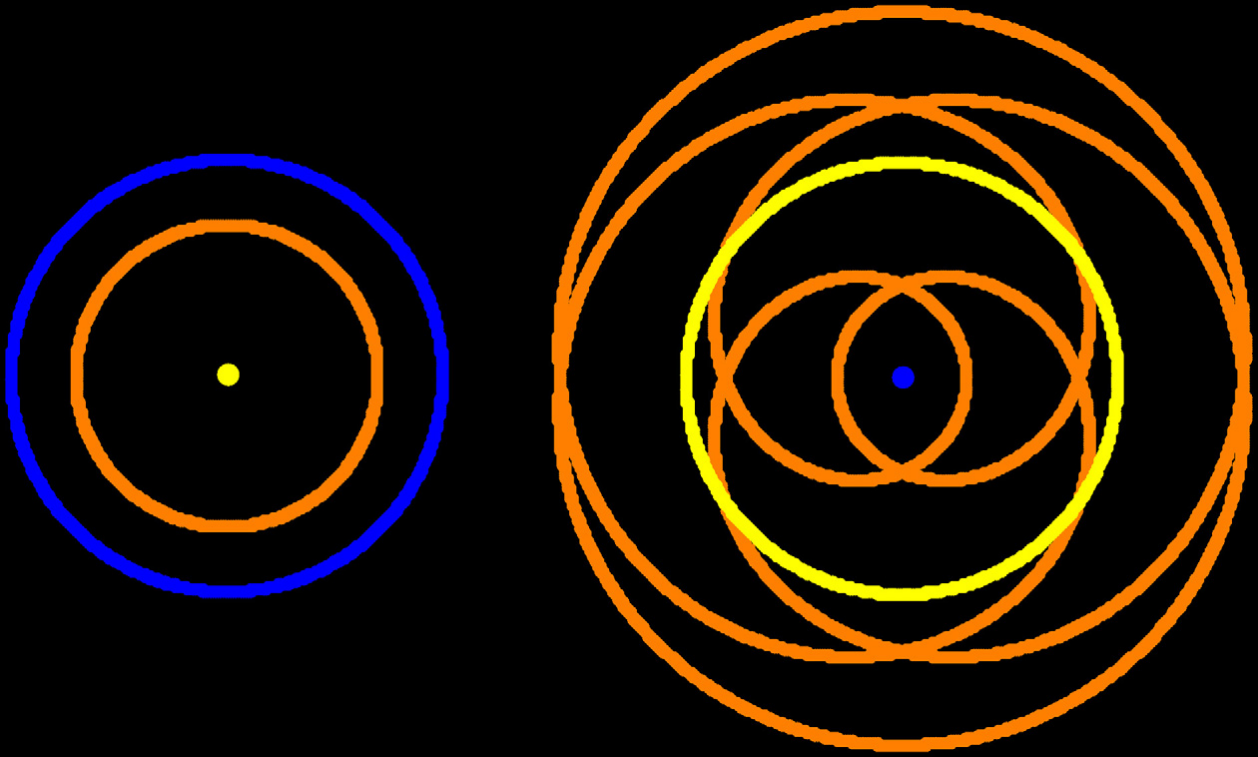
**Simplistic schematic of the heliocentric model (left) and the geocentric model (right)**. The motion of the Sun (yellow) and the planets Earth (blue) and Venus (orange) is shown. Under the heliocentric model both Earth and Venus have simple circular orbits around the Sun. In contrast, under the geocentric model Venus describes a complicated (albeit beautiful) path. The heliocentric model is by far the more parsimonious explanation for our observation of the motions of celestial bodies, especially when taking Newtonian physics into account. It is important to note that this heliocentric model is not the “true” model as for instance the orbits are not circular and the planets do not strictly revolve around the center of the sun. However, it is certainly the *better* model of the two as it requires fewer assumptions.

Certainly, science should be open-minded and no hypothesis must be dismissed out of hand—but this does not mean that every possible hypothesis is equally likely. A former colleague of mine once wrote very eloquently: “Science is not about finding the truth at all, but about finding better ways of being wrong” (Schofield, 2013). Not only does our present understanding fail to explain everything about the universe, we must accept that we will never explain everything. Importantly, this also means that we must always remain skeptical of *any* claims but especially our own.

It would be an easy mistake for “mainstream” researchers to accuse parapsychologists of being solely responsible for perpetuating this non-skeptical thinking. Yet it is only human to cling to theories against compelling evidence to the contrary. We all must be more critical. As Richard Feynman put it: “The first principle is that you must not fool yourself and you are the easiest person to fool” (Feynman, 2010). If some result is too good to be true, it probably is. We should actively strive for our hypotheses to be proven incorrect. And we should stop relying on statistics at the expense of objective reasoning. If we do not, I predict we will see many more such studies (on psi or something else) published in major science journals. They will not bring us any closer to understanding the cosmos.

## Conflict of interest statement

The author declares that the research was conducted in the absence of any commercial or financial relationships that could be construed as a potential conflict of interest.
